# Location of Femoral Fractures in Patients with Different Weight Classes in Fall and Motorcycle Accidents: A Retrospective Cross-Sectional Analysis

**DOI:** 10.3390/ijerph15061082

**Published:** 2018-05-27

**Authors:** Meng-Wei Chang, Hang-Tsung Liu, Chun-Ying Huang, Peng-Chen Chien, Hsiao-Yun Hsieh, Ching-Hua Hsieh

**Affiliations:** 1 Department of Emergency Medicine, Kaohsiung Chang Gung Memorial Hospital and Chang Gung University College of Medicine, Kaohsiung 833, Taiwan; infenit3868@gmail.com; 2 Department of Trauma Surgery, Kaohsiung Chang Gung Memorial Hospital and Chang Gung University College of Medicine, Kaohsiung 833, Taiwan; htl1688@yahoo.com.tw (H.-T.L.); junyinhaung@yahoo.com.tw (C.-Y.H.); 3 Department of Plastic Surgery, Kaohsiung Chang Gung Memorial Hospital and Chang Gung University College of Medicine, Kaohsiung 833, Taiwan; venu_chien@hotmail.com (P.-C.C.); sylvia19870714@hotmail.com (H.-Y.H.)

**Keywords:** fall, femoral fracture, motorcycle, obese, overweight, trauma, underweight

## Abstract

Background: This study aimed to determine the incidence of femoral fracture location in trauma patients with different weight classes in fall and motorcycle accidents. Methods: A total of 2647 hospitalized adult patients with 2760 femoral fractures from 1 January 2009 to 31 December 2014 were included in this study. Femoral fracture sites were categorized based on their location: proximal femur (type A, trochanteric; type B, neck; and type C, head), femoral shaft, and distal femur. The patients were further classified as obese (body mass index [BMI] of ≥30 kg/m^2^), overweight (BMI of <30 but ≥25 kg/m^2^), normal weight (BMI of <25 but ≥18.5 kg/m^2^), and underweight (BMI of <18.5 kg/m^2^). Odds ratios and 95% confidence intervals of the incidences of femoral fracture location were calculated in patients with different weight classes in fall or motorcycle accidents, and they were then compared with those in patients with normal weight. *p* values of <0.05 were considered statistically significant. Results: Most of the fractures sustained in fall accidents presented in the proximal type A (41.8%) and type B (45.3%) femur, whereas those sustained in motorcycle accidents involved the femoral shaft (37.1%), followed by the distal femur (22.4%) and proximal type A femur (21.2%). In fall accidents, compared with normal-weight patients, obese and overweight patients sustained lower odds of risk for proximal type B fractures but higher odds of risk for femoral shaft and distal femoral fractures. In motorcycle accidents, compared with normal-weight patients, obese patients sustained lower odds of risk for proximal type B fractures but no difference in odds of risk for femoral shaft and distal femoral fractures. Overweight and underweight patients who sustained fractures in a motorcycle accident did not have different fracture location patterns compared with normal-weight patients. Conclusions: This study revealed that femoral fracture locations differ between fall and motorcycle accidents. Moreover, greater soft tissue padding may reduce impact forces to the greater trochanteric region in obese patients during fall accidents, and during motorcycle accidents, the energy transmitted and the point of impact may dominantly determine the location of femoral fractures.

## 1. Background

Many studies have reported that obesity is associated with an increased risk for musculoskeletal injuries [1,2,3]. In children, extremely obese patients had a 1.45-fold increased risk for lower extremity fracture than normal-weight patients, and moderately obese and overweight patients had a 23% and 17% increased fracture risk, respectively [4]. This risk for lower extremity fracture increased with increasing weight classes in both men and women [4]. The GLOW study, a prospective multinational study that included 60,393 women aged ≥55 years [5], confirmed that obese patients had a higher frequency of falls [6] and were more likely to have experienced previous lower extremity fractures than other patients.

In lower extremity fractures, femoral fracture is associated with considerable morbidity [7,8]. Nonetheless, the incidence of femoral fracture is a complex phenomenon. Heavier patients are generally less active and may have fewer trauma tendencies; however, with increasing body weight, their feet may become more unstable. Obese people have increased bone density and are non-osteoporotic [9,10]. The potential energy associated with falls from a standing height impacting on the hip is greater than the average energy required to fracture an elderly hip [11]. As the body mass index (BMI) increases, the cross-sectional area, section modulus, and bone mineral density (BMD) of the femur increases [6]. However, the probability of fracture depends not only on its likelihood of occurring and on the bone strength but also on the force of impact during trauma. The magnitudes of traumatic impact forces increase in proportion to the body weight. Therefore, greater impact forces should increase the incidence of femoral fractures [12]. However, many studies reported that hip fracture rates were low with increasing BMI [6,13], which is thought to be due to increased padding provided by increased fat deposits over the trochanter and iliac wing areas [14,15].

Most of the studies on femoral fractures focus on patients sustaining a fall and less from other kinds of trauma, and studies on the location of femoral fractures have been limited. In Taiwan, motorcycles are a popular means of transportation and are a major reason for trauma in the population [16,17,18], leading to an increased incidence of motorcycle-related injuries and fatalities [19]. Obese motorcycle riders reportedly have different injury characteristics and patterns to normal-weight motorcycle riders [20,21]. Because the energy involved during an impact is directly proportional to both mass and velocity (squared), unrestrained individuals are at a higher risk for injury. An elevated BMI may dissipate high energy in a crash, thereby increasing the vulnerability of the victim to serious injury [22]. However, studies on the incidence of femoral fracture in motorcycle accidents have been limited. Therefore, this study aimed to determine the incidence of femoral fracture location in trauma patients with different weight classes in motorcycle and fall accidents.

## 2. Methods

### Study Design

**Figure 1:**
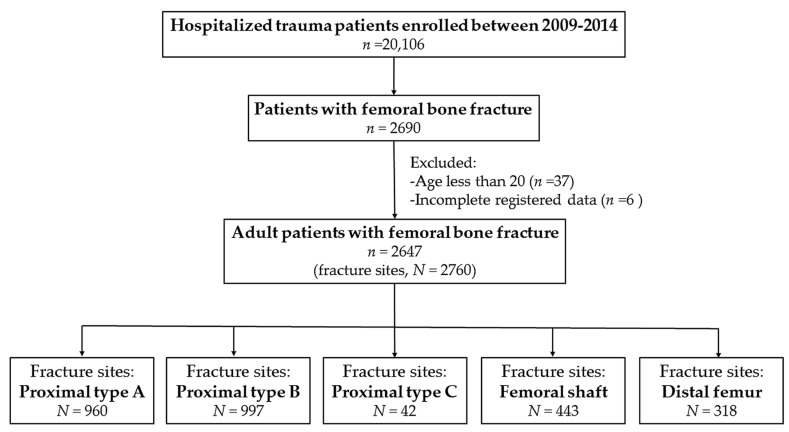
A flow chart presenting the grouping of fracture sites among the hospitalized adult patients with traumatic femoral bone fracture.

**Figure 2:**
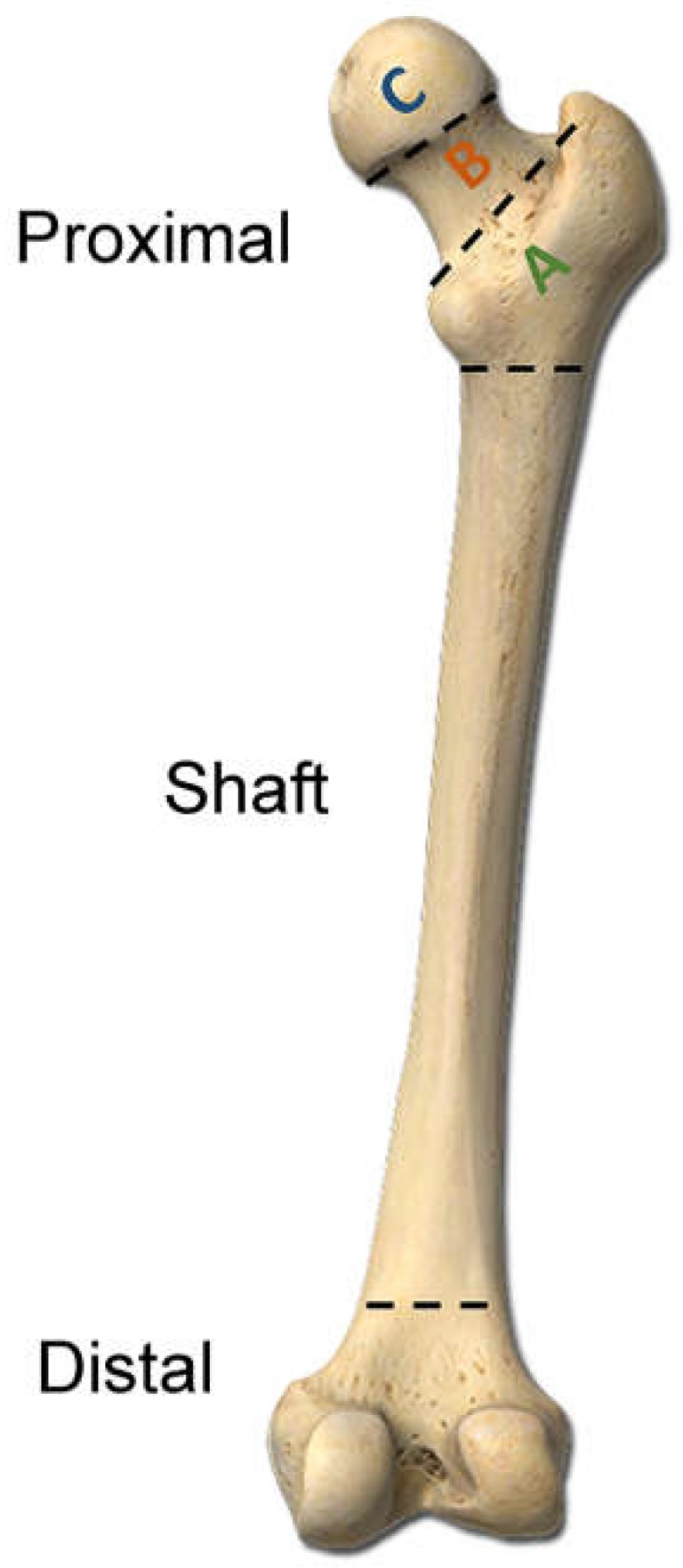
Types of femoral fracture according to their location in the proximal (type A, trochanteric; type B, neck; and type C, head), shaft, or distal femur based on the AO classification.

This study was approved by the Institutional Review Board (IRB) of the Chang Gung Memorial Hospital (approval number 105-1108C) before its implementation. According to IRB regulations, the need for informed consent was waived off. This retrospective study reviewed data of all 20,106 patients enrolled in the Trauma Registry System from 1 January 2009 to 31 December 2014 (Figure 1). The inclusion criteria were as follows: (1) adult patients aged ≥20 years and (2) hospitalization for the treatment of trauma with femoral fracture diagnoses. Patients with incomplete registered data were excluded. According to the World Health Organization’s definition [23,24], these trauma patients were categorized as obese (BMI of ≥30 kg/m^2^), overweight (BMI of <30 but ≥25 kg/m^2^), normal weight (BMI of <25 but ≥18.5 kg/m^2^), and underweight (BMI of <18.5 kg/m^2^). The retrieved patient data included age; sex; trauma mechanisms (fall from standing height, motorcycle accident, bicycle accident, motor vehicle accident, struck by/against an object, and pedestrian accident); BMI calculated as weight (kg)/height (m)^2^; Abbreviated Injury Scale (AIS) score of each body part; Injury Severity Score (ISS); and femoral fracture sites categorized according to their location as proximal femoral (type A:, trochanteric; type B, neck; and type C, head), femoral shaft, and distal femoral fractures based on the Arbeitsgemeinschaft für Osteosynthesefragen classification (Figure 2) [25]. The data collected were compared using IBM SPSS Statistics for Windows, version 22.0 (IBM Corp., Armonk, NY, USA). Odds ratio and 95% confidence intervals of the risk of a particular fracture location were calculated. *p* values of <0.05 were considered statistically significant.

## 3. Results

### 3.1. Characteristics of the Patients with Femoral Fracture

A total of 2647 patients with 2,760 femoral fractures were included in this study (Table 1), with 960 (34.8%) proximal femoral type A, 997 (36.1%) proximal femoral type B, 42 (1.5%) proximal femoral type C, 443 (16.1%) femoral shaft, and 318 (11.5%) distal femoral fractures. Among these patients, 1,153 (24.3%) and 1,494 (56.4%) were men and women, respectively, and 202 (7.6%) were obese, 643 (24.3%) were overweight, 1552 (58.6%) had normal weight, and 250 (9.4%) were underweight. Falling was the leading cause of femoral fractures (64.9%), followed by motorcycle (26.1%) and bicycle (4.2%) accidents. Associated injuries to the head/neck (8.7%), face (5.2%), thorax (4.9%), and abdomen (2.9%) were also found in these patients. With a median ISS of 9, a total of 2489 (94.0%), 81 (3.1%), and 77 (2.9%) patients had an ISS of <16, 16–24, and ≥25, respectively.

### 3.2. Location of Femoral Fractures in Patients with Different Injury Mechanisms

As shown in Table 2, most fractures sustained from a fall accident were proximal type A (41.8%) and type B (45.3%), whereas those in the motorcycle accident involved the femoral shaft (37.1%), followed by the distal femur (22.4%) and proximal type A (21.2%). Compared to patients who sustained fractures from a fall, those sustained fractures from a motorcycle accident had lower odds of proximal type A and proximal B fractures, but 17.2-fold higher odds of a proximal type C fracture, 8.7-fold of a femoral shaft fracture, and 4.2-fold of a distal femoral fracture; patients who sustained a motor vehicle accident had a fracture pattern similar to those who sustained fractures from motorcycle accidents, as evidenced by lower odds of proximal type A and B fractures, but 76.2-fold higher odds of proximal type C, 10.2-fold of femoral shaft, and 4.0-fold of distal femoral fractures; and patients who sustained an injury after being struck by/against an object also had a fracture pattern similar to those who sustained fractures from motorcycle accidents, as evidenced by lower odds of proximal type A and B fractures, but higher odds of femoral shaft and distal femoral fractures. Patients who sustained injuries as pedestrians had higher odds of femoral shaft and distal femoral fractures than those who sustained injury from a fall. In addition, differences in the location of femoral fracture between patients who had a fall accident and those who were injured via riding a bicycle was not observed.

### 3.3. Location of Femoral Fractures in Patients with Different Weight Classes in Fall Accidents

Among the patients who sustained fractures in a fall accident (Table 3), obese and overweight patients sustained 0.5-fold and 0.7-fold higher odds of proximal type B fractures, respectively, than those with normal weight. In addition, obese and overweight patients sustained 2.9-fold and 1.7-fold higher odds of femoral shaft fractures and 4.0-fold and 2.4-fold higher odds of distal femoral fractures than those with normal weight. In contrast, underweight patients presented 1.4-fold higher odds of a proximal type A fracture and 0.1-fold higher odds of a distal femoral fracture than those with normal weight.

### 3.4. Location of Femoral Fractures in Patients with Different Weight Classes in Motorcycle Accidents

Among the patients who sustained fractures from a motorcycle accident (Table 4), obese patients sustained a 0.4-fold higher odds of proximal type B fractures than the normal-weight patients. Moreover, overweight and underweight patients did not have a different fracture location pattern to normal-weight patients.

## 4. Discussion

This study revealed that the locations of femoral fractures differed between fall and motorcycle accidents; in the former, proximal type A and B fractures were predominant, and in the latter, femoral shaft fracture, followed by distal femoral and proximal type A fractures, were predominant. Similarly, the fracture location pattern was consistent in low-energy impact accidents such as bicycle and fall accidents, as well as in high-energy impact accidents such as motorcycle and motor vehicle accidents.

In fall accidents, compared with normal-weight patients, obese and overweight patients had lower odds of risk for proximal type B fractures but higher odds of risk for femoral shaft and distal femoral fractures. These results are in accordance with those of a study performed in a meta-analysis of 12 multinational cohorts of nearly 60,000 adults, showing that, independent of sex, patients with BMI of >25 kg/m^2^ had significantly lower rates of hip fractures than those with BMI of <25 kg/m^2^ [26]. When trauma occurs, the shape and structure of the femur determine how the forces are transmitted through the bone from the point of impact, which results in a fracture [27]. In a fall accident, the force directly impacts the posterolateral aspect of the greater trochanter, making the femoral neck particularly vulnerable to fractures [28]. However, although the magnitudes of traumatic impact forces increase in proportion to body weight and may result in increased incidences of femoral fractures, such increased incidences were only noted in the femoral shaft and distal femur and not in the proximal femur, as evidenced by decreased incidence of fractures in the femoral neck (type B) of obese patients. The most logical explanation is that the greater soft tissue padding in these sites compensates the greater impact forces that result from falls in obese patients [14,15]. The impact force disadvantage is more likely compensated by thicker soft tissue padding, which reduces the force transmitted to the bone.

In underweight patients, the adjusted BMD, cross-sectional area, and section modulus of the femur have been found to be lower than those with normal weight [6]. In this study, soft tissue padding should have little moderating effects on fall impact in underweight patients, as difference in the femoral type B fracture was not observed in underweight patients. Moreover, the fracture location pattern in underweight patients was quite different from that of obese patients, and the incidence of proximal type A femoral fractures was even higher in underweight patients than in normal-weight patients; this result was in agreement with the observation that the rate of hip fractures was twice as high in underweight patients than those in normal-weight patients [6].

The impact of energy transmitted to the bone is generally greater in motorcycle accidents than in fall accidents, and the point of impact to the femur is not limited to the greater trochanter, which is commonly observed in fall accidents. A similar scenario was found in motor vehicle accidents, demonstrating that elevated BMI increased the risk for lower-extremity injury in frontal crashes, but decreased the risks for injury in nearside impacts [14,15]. In this study, the odds of proximal type B fractures in obese patients was lower than those in normal-weight patients in motorcycle accidents. The protection effect of soft tissue padding was still found in obese but not in overweight patients, implying its protection effect may not be enough to offset the high energy impact transmitted during motorcycle accidents. In this study, overweight and underweight patients who sustained motorcycle accidents did not have a different fracture location pattern from those with normal weight, implying that the energy transmitted and the point of impact may dominantly determine the location of femoral fractures during motorcycle accidents.

Some limitations of this study should be acknowledged. First, an inherent selection bias existed because of the retrospective design. Second, the lack of data regarding the impact speed and force in motorcycle accidents and the use of any other protective materials limit the interpretation of the analyzed data. Third, the statistical analysis may be underpowered, especially regarding the incidence of femoral type C fracture, due to a small number of patients. Fourth, the population included in this study is limited to a single urban trauma center in southern Taiwan, which may not be representative of other populations. The lack of any information regarding the status of BMD, serum levels of calcium and vitamin D, and the medical/pharmacological history represent one important limitation of the study. Further, a bias may exist during the assessment of the relative risk for femoral fracture in different locations because the osteoporotic condition of the femur was unknown as patients who sustained a fall injury are generally older than those injured in a motorcycle accident.

## 5. Conclusions

This study revealed that the locations of femoral fracture differ between fall and motorcycle accidents. In fall accidents, compared with normal-weight patients, obese and overweight patients had lower odds of risk for proximal type B fractures but higher odds of risk for femoral shaft and distal femoral fractures. In motorcycle accidents, compared with normal-weight patients, obese patients had lower odds of risk for proximal type B fractures and no difference in odds of risk for femoral shaft and distal femoral fractures. This study implies that greater soft tissue padding may reduce the impact forces to the greater trochanteric region in obese patients during fall accidents, and the energy transmitted and the point of impact during motorcycle accidents may dominantly determine the location of femoral fractures.

## Figures and Tables

**Table 1:** Characteristics of patients with femoral fractures.

Variables	Patients *n* = 2647
**Gender, *n* (%)**	
**Male**	1153 (43.6)
**Female**	1494 (56.4)
**Age (years)**	66.5±19.5
**BMI classification, *n* (%)**	
**Obese**	202 (7.6)
**Overweight**	643 (24.3)
**Normal**	1552 (58.6)
**Underweight**	250 (9.4)
**Mechanisms, *n* (%)**	
**Fall**	1719 (64.9)
**Motorcycle**	690 (26.1)
**Bicycle**	112 (4.2)
**Motor vehicle**	54 (2.0)
**Struck by/against**	44 (1.7)
**Pedestrian**	28 (1.1)
**AIS, *n* (%)**	
**Head/Neck**	229 (8.7)
**Face**	137 (5.2)
**Thorax**	130 (4.9)
**Abdomen**	78 (2.9)
**ISS, median (IQR)**	9 (9–9)
**<16**	2489 (94.0)
**16–24**	81 (3.1)
**≥25**	77 (2.9)

**Table 2:** Location of femoral fracture in patients with different injury mechanisms.

Variables	Fall *n* = 1765 (I)	Motorcycle *n* = 744 (II)	Bicycle *n* = 113 (III)	Motor Vehicle *n* = 61 (IV)	Struck by/against *n* = 48 (V)	Pedestrian *n* = 29 (VI)
**Proximal-A**	737 (41.8)	158 (21.2)	40 (35.4)	8 (13.1)	9 (18.8)	8 (27.6)
**Proximal-B**	799 (45.3)	115 (15.5)	57 (50.4)	6 (9.8)	12 (25.0)	8 (27.6)
**Proximal-C**	4 (0.2)	28 (3.8)	1 (0.9)	9 (14.8)	0 (0.0)	0 (0.0)
**Shaft**	112 (6.3)	276 (37.1)	6 (5.3)	25 (41.0)	18 (37.5)	6 (20.7)
**Distal**	113 (6.4)	167 (22.4)	9 (8.0)	13 (21.3)	9 (18.8)	7 (24.1)

**Table:** 

Variables	*OR (95%CI)*	*p*	*OR (95%CI)*	*p*	*OR (95%CI)*	*p*	*OR (95%CI)*	*p*	*OR (95%CI)*	*p*
	II vs. I		III vs. I		IV vs. I		V vs. I		VI vs. I	
**Proximal-A**	0.4 (0.31–0.46)	<0.001	0.8 (0.51–1.14)	0.183	0.2 (0.10–0.45)	<0.001	0.3 (0.16–0.67)	0.001	0.5 (0.23–1.21)	0.125
**Proximal-B**	0.2 (0.18–0.28)	<0.001	1.2 (0.84–1.80)	0.284	0.1 (0.06–0.31)	<0.001	0.4 (0.21–0.78)	0.005	0.5 (0.20–1.05)	0.058
**Proximal-C**	17.2 (6.02–49.26)	<0.001	3.9 (0.44–35.46)	0.267	76.2 (22.73–255.44)	<0.001	—	1.000	—	1.000
**Shaft**	8.7 (6.83–11.09)	<0.001	0.8 (0.36–1.93)	0.660	10.2 (5.94–17.68)	<0.001	8.9 (4.79–16.38)	<0.001	3.9 (1.54–9.65)	0.010
**Distal**	4.2 (3.27–5.47)	<0.001	1.3 (0.62–2.57)	0.514	4.0 (2.08–7.52)	<0.001	3.4 (1.60–7.14)	0.004	4.7 (1.95–11.12)	0.002

**Table 3:** Location of femoral fracture in patients with different weight classes in fall accidents.

Variables	Obese *n* = 108 (II)	Overweight *n* = 415 (III)	Underweight *n* = 185 (IV)	Normal *n* = 1057 (I)	*OR (95%CI)*	*p*	*OR (95%CI)*	*p*	*OR (95%CI)*	*p*
					II vs. I		III vs. I		IV vs. I	
**Proximal-A**	39 (36.1)	170 (41.0)	91 (49.2)	437 (41.3)	0.8 (0.53–1.21)	0.292	1.0 (0.78–1.24)	0.894	1.4 (1.00–1.88)	0.046
**Proximal-B**	36 (33.3)	162 (39.0)	87 (47.0)	514 (48.6)	0.5 (0.35–0.80)	0.002	0.7 (0.54–0.85)	0.001	0.9 (0.69–1.28)	0.688
**Proximal-C**	0 (0.0)	3 (0.7)	0 (0.0)	1 (0.1)	—	1.000	7.7 (0.80–74.13)	0.070	—	1.000
**Shaft**	15 (13.9)	36 (8.7)	6 (3.2)	55 (5.2)	2.9 (1.60–5.40)	<0.001	1.7 (1.12–2.68)	0.013	0.6 (0.26–1.44)	0.255
**Distal**	18 (16.7)	44 (10.6)	1 (0.5)	50 (4.7)	4.0 (2.26–7.20)	<0.001	2.4 (1.57–3.64)	<0.001	0.1 (0.02–0.80)	0.008

**Table 4:** Location of femoral fracture in patients with different weight classes in motorcycle accidents.

Variables	Obese *n* = 92 (II)	Overweight *n* = 206 (III)	Underweight *n* = 47 (IV)	Normal *n* = 399 (I)	*OR (95%CI)*	*p*	*OR (95%CI)*	*p*	*OR (95%CI)*	*p*
					II vs. I		III vs. I		IV vs. I	
**Proximal-A**	19 (20.7)	40 (19.4)	12 (25.5)	87 (21.8)	0.9 (0.53–1.63)	0.809	0.9 (0.57–1.31)	0.494	1.2 (0.61–2.47)	0.561
**Proximal-B**	7 (7.6)	25 (12.1)	12 (25.5)	71 (17.8)	0.4 (0.17–0.86)	0.016	0.6 (0.39–1.04)	0.071	1.6 (0.78–3.20)	0.197
**Proximal-C**	5 (5.4)	9 (4.4)	1 (2.1)	13 (3.3)	1.7 (0.59–4.91)	0.353	1.4 (0.57–3.23)	0.489	0.6 (0.08–5.05)	1.000
**Shaft**	34 (37.0)	80 (38.8)	18 (38.3)	144 (36.1)	1.0 (0.65–1.66)	0.876	1.1 (0.80–1.59)	0.508	1.1 (0.59–2.05)	0.766
**Distal**	27 (29.3)	52 (25.2)	4 (8.5)	84 (21.1)	1.6 (0.94–2.59)	0.086	1.3 (0.85–1.88)	0.242	0.3 (0.12–1.02)	0.051
